# Gas Kinetic Scheme Coupled with High-Speed Modifications for Hypersonic Transition Flow Simulations

**DOI:** 10.3390/e26020173

**Published:** 2024-02-18

**Authors:** Chengrui Li, Wenwen Zhao, Hualin Liu, Youtao Xue, Yuxin Yang, Weifang Chen

**Affiliations:** 1 School of Aeronautics and Astronautics, Zhejiang University, Hangzhou 310027, China; lichengrui@zju.edu.cn (C.L.); xyt1215@zju.edu.cn (Y.X.); 12324007@zju.edu.cn (Y.Y.); chenwfnudt@zju.edu.cn (W.C.); 2 Huanjiang Laboratory, Shaoxing 311800, China; 3 College of Sciences, China Jiliang University, Hangzhou 310018, China; hualinliu@cjlu.edu.cn

**Keywords:** boundary layers, transition flow, gas kinetic scheme, hypersonic flow, turbulence models

## Abstract

The issue of hypersonic boundary layer transition prediction is a critical aerodynamic concern that must be addressed during the aerodynamic design process of high-speed vehicles. In this context, we propose an advanced mesoscopic method that couples the gas kinetic scheme (GKS) with the Langtry–Menter transition model, including its three high-speed modification methods, tailored for accurate predictions of high-speed transition flows. The new method incorporates the turbulent kinetic energy term into the Maxwellian velocity distribution function, and it couples the effects of high-speed modifications on turbulent kinetic energy within the computational framework of the GKS solver. This integration elevates both the transition model and its high-speed enhancements to the mesoscopic level, enhancing the method’s predictive capability. The GKS-coupled mesoscopic method is validated through a series of test cases, including supersonic flat plate simulation, multiple hypersonic cone cases, the Hypersonic International Flight Research Experimentation (HIFiRE)-1 flight test, and the HIFiRE-5 case. The computational results obtained from these cases exhibit favorable agreement with experimental data. In comparison with the conventional Godunov method, the new approach encompasses a broader range of physical mechanisms, yielding computational results that closely align with the true physical phenomena and marking a notable elevation in computational fidelity and accuracy. This innovative method potentially satisfies the compelling demand for developing a precise and rapid method for predicting hypersonic boundary layer transition, which can be readily used in engineering applications.

## 1. Introduction

The transition from laminar to turbulent flow is one of the few fundamental subject problems left over from classical mechanics. This phenomenon has considerable effects on essential physical parameters such as skin friction, heat transfer, noise, and mixing. Specifically, it is widely known that skin friction and heat flow levels can be estimated at around three to five times higher compared with laminar flow in turbulent flow [1,2]. Such differences are anticipated to be significantly amplified at hypersonic velocities, with potential implications for hypersonic vehicles’ aerodynamic performance and thermal protection systems. In the context of aerospace vehicles, the National Aerospace Plane (NASP) program was conducted to investigate the effects of boundary-layer transition on their performance [3]. The results of this program revealed that if the boundary layer were to remain in a fully laminar state instead of transitioning to a fully turbulent regime, the payload-to-gross-weight ratio for the aircraft would increase almost twofold. These findings underscore the importance of further research into this fundamental physical problem and the development of efficient hypersonic transition prediction methods to effectively address the associated design and engineering challenges.

Over the years, significant progress has been made in developing and refining numerical methods for predicting boundary-layer transition, empowering engineers to make well-informed design decisions. Among these methods, direct numerical simulation (DNS) stands out as a high-fidelity approach capable of modeling the entire flow field, resolving all scales, and accurately capturing the nonlinear disruption of boundary layer perturbations. DNS has demonstrated its effectiveness in capturing transitions in complex flows and providing valuable insights into the transition process. In a study by Li et al. [4,5], direct numerical simulations were performed to investigate the hypersonic boundary layer transition over a blunt cone and a lifting body at small angles of attack. The authors analyzed DNS data along with the underlying mechanisms driving transition flow. Another numerical approach, known as large eddy simulation (LES) [6], offers an advanced methodology that aims to simulate and solve for large-scale turbulent structures while modeling the impact of unresolved small-scale structures. Both DNS and LES face computational challenges due to the need for fine grid resolutions to resolve the smallest turbulent scales and accurately capture flow characteristics. The high-resolution requirements demand substantial computational resources and prolonged simulation periods, rendering these methods impractical for various engineering applications.

Numerical models based on the Reynolds-averaged Navier–Stokes (RANS) framework have emerged as a cost-effective and robust choice for simulating boundary layer transition, offering comparable accuracy and dispersion to LES under small angles of attack. The significance of turbulence/transition models in the field is evident, with approximately ten Reynolds-averaged RANS models proposed to date. Among these models, the classical four-equation $\gamma \text{–}Re_{\theta t}$ transition model introduced by Langtry and Menter [7,8] has demonstrated exceptional accuracy in predicting boundary layer transitions at low velocities. Notably, the $\gamma \text{–}Re_{\theta t}$ model relies solely on local variables and utilizes the vorticity Reynolds number instead of the momentum thickness Reynolds number, facilitating seamless integration into parallel computational fluid dynamics (CFD) codes. Consequently, it has garnered attention and widespread adoption within the computational fluid dynamics community [9,10,11], with renowned commercial CFD software packages incorporating the Langtry–Menter transition model, affirming its practical applicability in industrial settings. However, when the incoming flow reaches hypersonic velocities, the original Langtry–Menter transition model faces challenges in accurately predicting transition onset due to compressibility issues and the presence of multiple instability modes. To address these challenges, many scholars have devoted themselves to the research and development of the Langtry–Menter transition model for innovation and application in hypersonic boundary layer transition flows. You et al. [12] implemented the Langtry–Menter transition model in the DLR-TAU code for hypersonic flow cases, which accurately predicted hypersonic transition for blunted double-ramp cases but encountered overestimation of heat flux for shock impingement cases with sharp leading edges, indicating the need for further investigations. Zangeneh [13] evaluated the model’s performance in predicting boundary layer transition at hypersonic speeds and validated its potential for transition flow prediction in the hypersonic region. Yan et al. [14,15] improved the model by presenting a new correlation of momentum thickness Reynolds number, modifying the transition onset control function, and adjusting relevant model parameters to predict heat transfer accurately in hypersonic boundary layer transition. Wang et al. [16] developed an improved k–ω–γ model that accounted for the effects of different instability modes associated with variations in Mach numbers. Zhou et al. [17] enhanced the transition model for three-dimensional hypersonic flow by incorporating novel criteria for streamwise instability and crossflow instability, respectively. Frauholz et al. [18] successfully coupled the Langtry–Menter $\gamma \text{–}Re_{\theta t}$ transition model with Eisfeld’s Reynolds stress turbulence model (RSM) [19] and validated it using hypersonic flat plate and double-ramp test cases. Xia et al. [20] introduced a simplified local correlation-based transition model by removing the momentum thickness Reynolds number equation from the original $\gamma \text{–}Re_{\theta t}$ model and incorporating new correlations for the transition length function and critical Reynolds number, demonstrating its validation across low-speed to hypersonic flows with reduced computational cost. Furthermore, Zhang et al. [21,22] enhanced the performance of the $\gamma \text{–}Re_{\theta t}$ model for predicting hypersonic boundary layer transition by recalibrating empirical correlations, adjusting the turbulent Prandtl number, and incorporating a crossflow transition criterion inferred from hypersonic experimental data. Xiang et al. [23,24] formulated a localized hypersonic crossflow transition criterion derived from extended hypersonic transition data and integrated it into the modified $\gamma \text{–}Re_{\theta t}$ transition model for predictions of crossflow transition in hypersonic cases. Additionally, Zhang et al. [25] used a data-driven framework utilizing field inversion and machine learning techniques to extend the model’s prediction capability, resulting in improved accuracy in predicting hypersonic boundary layer transition, including onset location determination and transition zone length estimation. In recent years, the scientific community has witnessed the emergence of machine learning (ML) techniques and data-driven approaches as a contemporary and rapidly advancing research frontier in the investigation of turbulence and transition models. The proliferation of scholarly articles in this domain underscores the growing significance of this field, as evidenced by the 2017 Michigan Turbulence Modeling Symposium [26], which identified it as a focal point for intensive research. In July 2022, the Symposium on Turbulence Modeling convened in Virginia, gathering over 80 esteemed experts from educational institutions, government bodies, and various industries. This esteemed gathering served as a venue for deliberating on the pivotal aspects of turbulence and transition modeling while assessing the relevant outcomes derived from data-driven methodologies and ML techniques [27]. Fundamentally speaking, there is little essential difference between the calibration methods for modifying parameters, control functions, and empirical formulas in turbulence models using data-driven approaches and relying on human experience.

In addition to the conventional methodologies discussed earlier, an alternative approach has emerged to enhance the predictive capabilities of the Langtry–Menter transition model. This alternative perspective proposes coupling the transition model with the gas kinetic scheme (GKS) method. The GKS [28], a novel CFD algorithm, has been proposed and developed over the last few decades, demonstrating robustness and accuracy across a wide range of flow velocities in simulation studies [29]. The GKS method proves to be more promising in addressing the challenges associated with hypersonic flow compared with traditional CFD algorithms, to some extent [30,31]. For instance, it has exhibited exceptional performance in accurately calculating heat flux and pressure loads in hypersonic flows [32]. Liu and Shu et al. [33,34] developed two high-order hybrid methods utilizing unstructured grids, specifically, the Least Square-based Finite Difference–Finite Volume (LSFD-FV) method and the Implicit Radial Basis Function-based Differential Quadrature–Finite Volume (IRBFDQ-FV) method. These methods were applied to simulate 3D incompressible and compressible flows, including hypersonic viscous flow. In both approaches, the gas kinetic flux solver was used for the simultaneous evaluation of inviscid and viscous fluxes in compressible viscous flow. The obtained results demonstrate that these methods exhibit higher accuracy compared with conventional methods using the same mesh. Researchers have extensively investigated the efficacy of the gas kinetic scheme for conducting DNS and LES of isotropic compressible turbulence, extending into the supersonic regime [31,35,36,37]. Some innovative studies have also been performed on GKS with turbulence models. Righi [38,39] combined the k–ω turbulence model with the gas kinetic scheme, yielding promising results in benchmark cases involving significant shock–boundary layer interactions. Tan et al. [40] developed an extended GKS for turbulence simulations based on the generalized Bhatnagar–Gross–Krook (BGK) [41] equation, using an effective relaxation time derived from turbulent viscosity, thus enabling the direct combination of several turbulence models. Zhong et al. [42] presented the development of a second-order gas kinetic scheme coupled with the Shear Stress Transport (SST) two-equation turbulence model for simulating compressible and incompressible turbulent flows. Cao et al. [43] focused on high-Reynolds number engineering flows and developed an implicit high-order gas kinetic scheme combined with the k–ω SST model for turbulent flow simulations. It should be noted that prior researchers used the GKS solver in combination with turbulence models, which is a loose coupling approach similar to the frequently used RANS solver. Liu et al. [44] took a different approach and developed a strongly coupled method, incorporating the k–ω SST turbulence model into the GKS by considering turbulent kinetic energy in the Maxwellian distribution function. These endeavors have pioneered new directions and research strategies that leverage the gas kinetic scheme to enhance turbulence modeling. By combining the strengths of the GKS and turbulence models, researchers aim to achieve more accurate simulations of complex flows.

In this manuscript, we present a closed-loop integration of the Langtry–Menter transition model and its high-speed modification methods with the GKS solver. This integration is accomplished with the direct or indirect influence of high-speed modification methods on the turbulent kinetic energy source term, as well as by incorporating turbulent kinetic energy into the Maxwellian distribution function. By combining these components, we effectively leverage their individual strengths and develop a mesoscopic numerical methodology capable of accurately predicting high-speed transitional flows. The subsequent sections of this paper are structured as follows: In Section 2, we provide a comprehensive overview of the mesoscopic transition prediction method framework, encompassing the gas kinetic scheme, the Langtry–Menter transition model, and three high-speed modification methods. We present a detailed derivation and description of each module. Section 3 elucidates the coupling mechanism inherent in the proposed method, elucidating the interaction mechanisms among the three core modules. Moving on to Section 4, we validate the proposed method through an extensive series of test cases, including a supersonic adiabatic flat plate, multiple hypersonic cone cases, the Hypersonic International Flight Research Experimentation (HIFiRE)-1 flight test, and the HIFiRE-5 case. A comparative analysis and discussion of the results obtained from the mesoscopic GKS method and the conventional Godunov method are conducted, with experimental data serving as a reference. Finally, in Section 5, we offer a comprehensive summary of the findings and contributions presented in this paper.

## 2. Mesoscopic Transition Prediction Method Framework

This manuscript presents a novel numerical framework that incorporates mesoscopic considerations. The framework comprises three key modules: the GKS solver module, the Langtry–Menter transition model module, and the high-speed correction method module.

### 2.1. Gas Kinetic Scheme

The gas kinetic scheme is a statistical theory that revolves around the Boltzmann equation and its simplified model, the BGK model. The Boltzmann equation, which describes the behavior of a single-component monatomic gas in the absence of external forces, forms the central foundation of this scheme.

(1) $$\frac{\partial f}{\partial t}+u_{i}\frac{\partial f}{\partial x_{i}}=Q\left(f,f\right)$$

where i=1,2,3 represents the three directions, $x_{i}$ represents the spatial variable, $u_{i}$ reprensents the particle velocity, f=f(x,t,u) is the gas distribution function, and Q(f,f) is the collision term used to describe the binary collision. The collision term at the right-hand side of the Boltzmann equation is simplified into a relaxation term to obtain the BGK model equation as follows:

(2) $$\frac{\partial f}{\partial t}+u_{i}\frac{\partial f}{\partial x_{i}}=\frac{g-f}{\tau }$$

where τ is particle collision time and g is equilibrium Maxwellian distribution function expressed as follows:

(3) $$g=\rho {\left(\frac{\lambda }{\pi }\right)}^{\frac{N+3}{2}}e^{-\lambda \left({\left(u_{i}-U_{i}\right)}^{2}+\xi^{2}\right)}$$

Referring to Equations (2) and (3), the BGK model governing equation for the current method is:

(4) $$\frac{\partial f}{\partial t}+u_{i}\frac{\partial f}{\partial x_{i}}=\frac{g-f}{\tau +\tau_{t}}$$

where τ represents laminar flow collision times and the extra term $\tau_{t}$ represents turbulent collision times calculated by the formulas:

(5) $$\tau =\frac{\mu }{p}\;\mathrm{and}\;\tau_{t}=\frac{\mu_{t}}{p}$$

The calculation of laminar viscosity μ is based on Sutherland’s law, while the turbulent viscosity $\mu_{t}$ is derived from the turbulence model proposed by Langtry 7, which will be expounded upon subsequently. The extended Maxwellian distribution function can be expressed as:

(6) $$g=\rho {\left(\frac{\lambda }{\pi }\right)}^{\frac{N+3}{2}}e^{-\lambda \left({\left(u_{i}-U_{i}\right)}^{2}+\xi^{2}\right)}{\left(\frac{\lambda_{t}}{\pi }\right)}^{\frac{N_{t}}{2}}e^{-\lambda_{t}\xi_{t}^{2}}$$

where ρ is the density, $U_{i}$ represents the macroscopic fluid velocity, and $u_{i}$ represents the microscopic velocity. $\lambda =\frac{m_{0}}{2k_{B}T}$, where $m_{0}$ is the molecular mass, $k_{B}$ is the Boltzmann constant and T is the temperature. $\lambda_{t}=\frac{m_{0}}{2k_{B}T_{t}}$, where $T_{t}$ represents the thermodynamic temperature equivalent to the turbulent kinetic energy. N is the total number of internal degrees of freedom and ξ denotes the internal variables. For three-dimensional equilibrium diatomic gas, N=2, which accounts for the two rotational modes. In the context of turbulent flow, $N_{t}$ represents the degrees of freedom of the internal energy that correspond to the turbulent kinetic energy. Consequently, the turbulent kinetic energy can be converted into internal energy and exhibit a strong coupling with the GKS methodology. The degrees of freedom of $N_{t}$ are fixed here as a constant of 3, which can be thought of as similar to three internal energy rotational modes. Upon comparing Equations (6) and (3), it can be observed that the additional part ${\left(\frac{\lambda_{t}}{\pi }\right)}^{\frac{N_{t}}{2}}e^{-\lambda_{t}\xi_{t}^{2}}$ is an extension to the Maxwellian distribution function, which is a bold attempt to achieve a strong coupling between the GKS method and the turbulence/transition model.

The macroscopic variables can be obtained by integrating the moments of the gas distribution function as follows:

(7) $$W=\left(\begin{array}{c} \rho \\ \begin{array}{l} \rho U\\ \rho E \end{array}\\ \rho E_{t} \end{array}\right)=\int \psi_{\alpha }fd\Xi$$

where W denotes the conservative variables, including density ρ, momentum ρU, and total energy ρE. $U={\left(U_{x},U_{y},U_{z}\right)}^{T}$ is the macroscopic velocity. $\psi_{\alpha }={\left(1,u,v,w,\frac{1}{2}\left(u^{2}+v^{2}+w^{2}+\xi^{2}+\xi_{t}^{2}\right),\frac{1}{2}\xi_{t}^{2}\right)}^{T}$ is the collision invariant vector after the introduction of turbulent kinetic energy as a non-conservative quantity and $d\Xi =dudvdwd\xi d\xi_{t}$ represents phase space. $\rho E_{t}$ denotes turbulent kinetic energy, which can be mathematically expressed as follows:

(8) $$\rho E_{t}=\int \frac{1}{2}\xi_{t}^{2}fd\Xi =\rho \frac{N_{t}}{4\lambda_{t}}=\rho \frac{N_{t}}{2}RT_{t}$$

namely:

(9) $$T_{t}=\frac{2E_{t}}{N_{t}R}$$

The compatibility condition can be written as:

(10) $$\int \psi_{\alpha }\frac{g-f}{\tau +\tau_{t}}d\Xi =\left(0,0,0,0,0,S_{\rho E_{t}}\right)$$

The calculation of the source term for turbulent kinetic energy $S_{\rho E_{t}}$ can be derived from the k–ω SST model.

The present study uses a finite volume method (FVM) to numerically solve the BGK-type model Equation (4) in order to verify the efficacy of the current GKS method for simulating hypersonic turbulent and transitional flows. The primary objective of the FVM’s GKS solver is to calculate the time-dependent gas distribution function at the grid interface, enabling the computation of numerical fluxes. This paper primarily focuses on the computation of fluxes for the variables involving $\left(\rho ,\rho U,\rho V,\rho W,\rho E,\rho E_{t}\right)$. By utilizing the formal integral solution of the BGK model in the local coordinate system, the construction of the gas distribution function f at the grid interface, denoted as in Equation (4), can be achieved as follows:

(11) $$\begin{array}{ll} f\left(x_{i+1/2},y_{j},z_{k},t,u,v,w,\xi \right)= & \frac{1}{\tau +\tau_{t}}\int_{0}^{t}g\left(x^{\prime },y^{\prime },z^{\prime },t^{\prime },u,v,w,\xi \right)e^{-\frac{t-t^{\prime }}{\tau +\tau_{t}}}dt^{\prime }\\ & +e^{-\frac{t}{\tau +\tau_{t}}}f_{0}\left(x_{i+1/2}-ut,y_{j}-vt,z_{k}-wt,\xi \right) \end{array}$$

where ${\left(u,v,w\right)}^{T}$ is the particle velocity and ${\left(x^{\prime },y^{\prime },z^{\prime }\right)}^{T}$ represents the trajectory of particles, namely, $x^{\prime }=x_{i+1/2}+u\left(t^{\prime }-t\right)$, $y^{\prime }=y_{j}+v\left(t^{\prime }-t\right)$ and $z^{\prime }=z_{j}+w\left(t^{\prime }-t\right)$. $t^{\prime }$ denotes the moment of motion of the particle, i.e., a point in the motion of the particle in the time period 0 to t. $f_{0}$ is the initial gas distribution function. The integral solution of Equation (11) characterizes the physical process of evolving from a kinetic scale to a hydrodynamic scale. Specifically, it describes the evolution of the particle-free transport behavior in $f_{0}$ to the macroscopic behavior of the hydrodynamic flow in the integral of g. The flow behavior at the cell interface depends on the ratio of the time step to the local particle collision time $\frac{\Delta t}{\tau +\tau_{t}}$. The hydrodynamic scale component, denoted as $\frac{1}{\tau }\int_{0}^{t}g\left(x^{\prime },y^{\prime },z^{\prime },t^{\prime },u,v,w,\xi \right)e^{-\frac{t-t^{\prime }}{\tau }}dt^{\prime }$, is a constituent of Equation (11), while the kinetic scale component, represented by $e^{-\frac{t}{\tau }}f_{0}\left(x_{i+1/2}-ut,y_{j}-vt,z_{k}-wt,\xi \right)$, constitutes another part of the equation. g is expanded as follows:

(12) $$g=g_{0}\left(1+\bar{a}x+\bar{b}y+\bar{c}z+\bar{A}t\right)$$

where $g_{0}$ is the initial equilibrium state distribution function over the center of the interface, which can be determined by applying the compatibility condition, resulting in the following equation:

(13) $$\int \psi_{\alpha }g_{0}d\Xi =W_{0}=\int_{u>0}\psi_{\alpha }g^{l}d\Xi +\int_{u<0}\psi_{\alpha }g^{r}d\Xi$$

The macroscopic values at the interface, denoted as $W_{0}$, are obtained from the reconstructed values of the left and right sides of the interface after right-hand and left-hand collisions, respectively. It can be seen from Equation (13) that $g_{0}$ is determined by the macroscopic values $W_{0}$ at the interface. $g^{l}$ and $g^{r}$ are Maxwellian distribution functions for the left and right sides of the cell interface, which are related to the macroscopic values reconstructed at the left and right sides of a cell interface. The initial gas distribution function $f_{0}$ in Equation (11) is constructed as follows:

(14) $$f_{0}=\{\begin{array}{c} g^{l}\left[\left(1+a^{l}x+b^{l}y+c^{l}z\right)-\left(\tau +\tau_{t}\right)\left(a^{l}u+b^{l}v+c^{l}w+A^{l}\right)\right],x\leq 0\\ g^{r}\left[\left(1+a^{r}x+b^{r}y+c^{r}z\right)-\left(\tau +\tau_{t}\right)\left(a^{r}u+b^{r}v+c^{r}w+A^{r}\right)\right],x>0 \end{array}$$

The coefficients denoted as $\bar{a},\;\bar{b},\;\bar{c},\;\bar{A},\;a^{l},\;a^{r},\;b^{l},\;b^{r},\;c^{l},\;c^{r},\;A^{l},\;A^{r}$ in Equations (12) and (14) represent the spatial and temporal derivatives of the Maxwellian distribution function, which are microscopic slopes obtained from Taylor expansions. In this instance, $\bar{a},\;a^{l},\;a^{r}$ represent the normal slopes, $\bar{b},\;b^{l},\;b^{r}$ denote the tangential slopes, and $\bar{c},\;c^{l},\;c^{r}$ refer to an additional set of tangential slopes oriented in the orthogonal direction. The coefficients mentioned above are derived from the Taylor expansion of the Maxwellian distribution function, which exhibits a dependence on the velocity of the particles in the following manners:

(15) $$\begin{array}{l} \bar{a}=\bar{a_{1}}+\bar{a_{2}}\cdot u+\bar{a_{3}}\cdot v+\bar{a_{4}}\cdot w+\bar{a_{5}}\cdot \frac{1}{2}\left(u^{2}+v^{2}+w^{2}+\xi^{2}\right)=\bar{a_{\alpha }}\psi_{\alpha }\\ \ldots \ldots \\ \bar{A}=\bar{A_{1}}+\bar{A_{2}}\cdot u+\bar{A_{3}}\cdot v+\bar{A_{4}}\cdot w+\bar{A_{5}}\cdot \frac{1}{2}\left(u^{2}+v^{2}+w^{2}+\xi^{2}\right)=\bar{A_{\alpha }}\psi_{\alpha } \end{array}$$

By examining the relationship between the distribution function and the macroscopic variables in Equation (7), we can derive the subsequent equations:

(16) $$\begin{array}{l} \int g^{l}\psi_{\alpha }d\Xi =W^{l}(x_{i+1/2},y_{j},z_{k})\\ \int g^{r}\psi_{\alpha }d\Xi =W^{r}(x_{i+1/2},y_{j},z_{k})\\ \int g^{l}a^{l}\psi_{\alpha }d\Xi =\vec{n}\cdot \nabla W^{l}\\ \int g^{r}a^{r}\psi_{\alpha }d\Xi =\vec{n}\cdot \nabla W^{r}\\ \int g^{l}b^{l}\psi_{\alpha }d\Xi =\vec{t_{1}}\cdot \nabla W^{l}\\ \int g^{r}b^{r}\psi_{\alpha }d\Xi =\vec{t_{1}}\cdot \nabla W^{r}\\ \int g^{l}c^{l}\psi_{\alpha }d\Xi =\vec{t_{2}}\cdot \nabla W^{l}\\ \int g^{r}c^{r}\psi_{\alpha }d\Xi =\vec{t_{2}}\cdot \nabla W^{r} \end{array}$$

where $\vec{n}$ represents the normal unit vector, while $\vec{t_{1}},\vec{t_{2}}$ denote the tangential unit vector. $W^{l}(x_{i+1/2},y_{j},z_{k})$ and $W^{r}(x_{i+1/2},y_{j},z_{k})$ are the reconstructed macroscopic variables on the left and right sides of the interface. $\nabla W^{l}$ and $\nabla W^{r}$ are the gradients of macroscopic variables on the left and right sides of the interface. These physical quantities are related as follows:

(17) $$W=\left\{ \begin{array}{l} W^{l}(x_{i+1/2},y_{j},z_{k})+\nabla W^{l}\cdot xx<0\\ W^{r}(x_{i+1/2},y_{j},z_{k})+\nabla W^{r}\cdot xx\geq 0 \end{array}\right.$$

The equations in (16) are simplified as follows:

(18) $$\begin{array}{l} M_{\alpha \beta }^{l,r}\cdot a_{\beta }^{l,r}=\frac{1}{\rho^{l,r}}\cdot \vec{n}\cdot \nabla W^{l,r}\\ M_{\alpha \beta }^{l,r}\cdot b_{\beta }^{l,r}=\frac{1}{\rho^{l,r}}\cdot {\vec{t}}_{1}\cdot \nabla W^{l,r}\\ M_{\alpha \beta }^{l,r}\cdot c_{\beta }^{l,r}=\frac{1}{\rho^{l,r}}\cdot {\vec{t}}_{2}\cdot \nabla W^{l,r} \end{array}$$

where $M_{\alpha \beta }^{l,r}=\frac{1}{\rho^{l,r}}\int g^{l,r}\psi_{\alpha }\psi_{\beta }d\Xi$. Microscopic slopes of the velocity distribution function at the interface, denoted as $\bar{a},\;\bar{b},\;\bar{c}$, can be determined by calculating the gradients of macroscopic physical quantities at the corresponding interface face centers as follows:

(19) $$\begin{array}{l} \int g_{0}\bar{a}\psi_{\alpha }d\Xi =\vec{n}\cdot \nabla \bar{W}\\ \int g_{0}\bar{b}\psi_{\alpha }d\Xi =\vec{t_{1}}\cdot \nabla \bar{W}\\ \int g_{0}\bar{c}\psi_{\alpha }d\Xi =\vec{t_{2}}\cdot \nabla \bar{W} \end{array}$$

The microscopic slopes of the velocity distribution function with respect to time, denoted as $A^{l},\;A^{r},\;\bar{A}$, can be obtained from the compatibility condition as follows:

(20) $$\begin{array}{l} \int \left(a^{l}u+b^{l}v+c^{l}w+A^{l}\right)\psi_{\alpha }g^{l}d\Xi =0\\ \int \left(a^{r}u+b^{r}v+c^{r}w+A^{r}\right)\psi_{\alpha }g^{r}d\Xi =0\\ \int \left(\bar{a}u+\bar{b}v+\bar{c}w+\bar{A}\right)\psi_{\alpha }g_{0}d\Xi =0 \end{array}$$

At this point, all the microscopic slopes can be solved. Substituting Equations (12) and (14) into Equation (11) yields an expression for the second-order gas distribution function at the interface, as follows:

(21) $$\begin{array}{l} f=\left(1-e^{-t/\tau }\right)g_{0}\\ +\left[\tau \left(-1+e^{-t/\tau }\right)+te^{-t/\tau }\right]\left[\bar{a}u+\bar{b}v+\bar{c}w\right]ug_{0}\\ +\left(t-\tau +\tau e^{-t/\tau }\right)\bar{A}g_{0}\\ +e^{-t/\tau }\left[H\left[u\right]g^{l}+\left(1-H\left[u\right]\right)g^{r}\right]\\ +e^{-t/\tau }\left(-t-\tau \right)\left[\left(ua^{l}+vb^{l}+wc^{l}\right)H\left[u\right]g^{l}+\left(ua^{r}+vb^{r}+wc^{r}\right)\left(1-H\left[u\right]\right)g^{r}\right]\\ +\left(-\tau \cdot e^{-t/\tau }\right)\left[A^{l}H\left[u\right]g^{l}+A^{r}\left(1-H\left[u\right]\right)g^{r}\right] \end{array}$$

where H[x] is the Heaviside function. For continuous flows, the finite volume method for updating macroscopic conservative variables within each control volume is calculated as follows:

(22) $$W_{i,j,k}^{n+1}=W_{i,j,k}^{n}-\frac{1}{\Delta V}\sum_{S}\int_{t^{n}}^{t^{n+1}}F_{W}\cdot n_{s}Sdt$$

where S is interface area, $n_{s}$ is the interface normal vector and $F_{W}$ is the interface flux. The difference between the GKS and conventional CFD is the method used to solve for the numerical flux. In the GKS solution method, the numerical flux F is obtained by solving for the moments of the velocity distribution function as follows:

(23) $$F_{W}=\int u\psi fd\Xi$$

That is:

(24) $$\left(\begin{array}{c} \begin{array}{c} F_{\rho }\\ F_{\rho u} \end{array}\\ F_{\rho v}\\ F_{\rho w}\\ F_{\rho E} \end{array}\right)=\int u\left(\begin{array}{c} 1\\ u\\ v\\ w\\ \frac{1}{2}\left(u^{2}+v^{2}+w^{2}+\xi^{2}+{\xi_{t}}^{2}\right) \end{array}\right)f\left(x_{i+1/2},y_{j},z_{k},t,u,v,w,\xi \right)d\Xi$$

The GKS solution process is complete.

### 2.2. Langtry–Menter Transition Model

The turbulence and transition models used in the current coupled method are the k–ω SST turbulence and $\gamma \text{–}Re_{\theta t}$ transition models. The $\gamma \text{–}Re_{\theta t}$ transition model encompasses two transport equations, namely, the intermittency factor γ transport equation, Equation (25), and the transport equation for the transition momentum thickness Reynolds number $\tilde{R}e_{\theta t}$, Equation (26).

(25) $$\frac{\partial (\rho \gamma )}{\partial t}+\frac{\partial \left(\rho U_{j}\gamma \right)}{\partial x_{j}}=P_{\gamma }-E_{\gamma }+\frac{\partial }{\partial x_{j}}\left[\left(\mu +\frac{\mu_{t}}{\sigma_{f}}\right)\frac{\partial \gamma }{\partial x_{j}}\right]$$

(26) $$\frac{\partial \left(\rho \tilde{R}e_{\theta t}\right)}{\partial t}+\frac{\partial \left(\rho U_{j}\tilde{R}e_{\theta t}\right)}{\partial x_{j}}=P_{\theta t}+\frac{\partial }{\partial x_{j}}\left[\sigma_{\theta t}\left(\mu +\mu_{t}\right)\frac{\partial \tilde{R}e_{\theta t}}{\partial x_{j}}\right]$$

The intermittency factor γ is used to initiate the transition and assumes a value of 0 for laminar flow and a value of 1 for fully turbulent flow. The production term, $P_{\gamma }$, and destruction term, $E_{\gamma }$, in Equation (25) are provided as:

(27) $$P_{\gamma }=F_{\mathrm{length}}c_{a1}\rho S{\left[\gamma F_{\mathrm{onset}}\right]}^{0.5}\left(1-c_{e1}\gamma \right)$$

(28) $$E_{\gamma }=c_{a2}\rho \Omega \gamma F_{\mathrm{turb}\;}\left(c_{e2}\gamma -1\right)$$

where $F_{length}$ is the transition region length control function, and $F_{onset}$ is designed to trigger the production of intermittency, which are dimensionless functions that play a crucial role in governing the intermittency factor transport equation within the boundary layer. $c_{a1},\;c_{e1},\;c_{a2}\;\mathrm{and}\;c_{e2}$ are model coefficients. The concept of the vorticity Reynolds number $Re_{v}$ is introduced into the $\gamma \text{–}Re_{\theta t}$ transition model and is a factor in the $F_{\mathrm{onset}}$ function, which is defined as follows:

(29) $$Re_{v}=\frac{\rho y^{2}}{\mu }S$$

where y is the distance to the wall and S is strain rate magnitude. The introduction of $Re_{v}$ localizes the numerical calculation and links the transition Reynolds number to local boundary layer properties. As the thickness of the boundary layer grows, the parameter $y^{2}S$ also increases and $Re_{v}$ gradually reaches a critical value to trigger the onset of transitions, thus determining the characteristics of the transition location for $F_{\mathrm{onset}}$. The boundary condition of the intermittency factor γ is 0 at the wall and 1 at the inflow entrance. The transition onset is controlled by the following functions:

(30) $$F_{\mathrm{onset}1}=\frac{Re_{v}}{2.193Re_{\theta c}}$$

(31) $$F_{\mathrm{onset}2}=\min\left[\max\left(F_{\mathrm{onset}1},F_{\mathrm{onset}1}^{4}\right),2.0\right]$$

(32) $$R_{T}=\frac{\rho k}{\mu \omega }$$

(33) $$F_{\mathrm{onset}3}=\max\left[1-{\left(\frac{R_{T}}{2.5}\right)}^{3},0\right]$$

(34) $$F_{\mathrm{onset}}=\max\left(F_{\mathrm{onset}2}-F_{\mathrm{onset}3},0\right)$$

where $Re_{\theta c}$ is the critical Reynolds number at which the intermittency in the boundary layer first begins to increase. Both $Re_{\theta c}$ and $F_{length}$ are empirical functions of $\tilde{R}e_{\theta t}$ with the following expressions:

(35) $$F_{\mathrm{length}}=\{\begin{array}{l} \left[398.189\cdot 10^{-1}+\left(-119.270\cdot 10^{-4}\right)\tilde{R}e_{\theta t}+\left(-132.567\cdot 10^{-6}\right)\tilde{R}{e_{\theta t}}^{2}\right],\tilde{R}e_{\theta t}<400\\ \left[\begin{array}{l} 263.404+\left(-123.939\cdot 10^{-2}\right)\tilde{R}e_{\theta t}+\left(194.548\cdot 10^{-5}\right)\tilde{R}{e_{\theta t}}^{2}\\ +\left(-101.695\cdot 10^{-8}\right)\tilde{R}{e_{\theta t}}^{3} \end{array}\right],400\leq \tilde{R}e_{\theta t}<596\\ \left[0.5-\left(\tilde{R}e_{\theta t}-596.0\right)\cdot 3.0\cdot 10^{-4}\right],596\leq \tilde{R}e_{\theta t}<1200\\ {}[0.3188],1200\leq \tilde{R}e_{\theta t} \end{array}$$

(36) $$Re_{\theta c}=\left\{ \begin{array}{l} \left[\tilde{R}e_{\theta t}-\left(\begin{array}{l} 396.035\cdot 10^{-2}+\left(-120.656\cdot 10^{-4}\right)\tilde{R}e_{\theta t}+\left(868.230\cdot 10^{-6}\right)\tilde{R}{e_{\theta t}}^{2}\\ +\left(-696.506\cdot 10^{-9}\right)\tilde{R}{e_{\theta t}}^{3}+\left(174.105\cdot 10^{-12}\right)\tilde{R}{e_{\theta t}}^{4} \end{array}\right)\right],\tilde{R}e_{\theta t}\leq 1870\\ \left[\tilde{R}e_{\theta t}-\left(593.11+\left(\tilde{R}e_{\theta t}-1870.0\right)\cdot 0.482\right)\right],\tilde{R}e_{\theta t}>1870 \end{array}\right.$$

The source term, denoted as $P_{\theta t}$ in Equation (26), serves to relate the transported scalar $\tilde{R}e_{\theta t}$ to $Re_{\theta t}$ derived from an empirical correlation outside the boundary layer, allowing for the diffusion of the transported scalar $\tilde{R}e_{\theta t}$ from the free stream. It is noteworthy that $\tilde{R}e_{\theta t}$ is the local transition onset momentum thickness Reynolds number obtained from the transport Equation (26) and $Re_{\theta t}$ is the transition onset momentum thickness Reynolds number derived from an empirical correlation based on freestream conditions. The source term $P_{\theta t}$ is represented as follows:

(37) $$P_{\theta t}=c_{\theta t}\frac{\rho }{t}\left(Re_{\theta t}-\tilde{R}e_{\theta t}\right)\left(1.0-F_{\theta t}\right)$$

The blending function $F_{\theta t}$ in the source term serves as a boundary layer identifier, with a value of 1 in the boundary layer and 0 in the freestream. This property turns the source term on outside the boundary layer and off inside the boundary layer, which implies that outside the boundary layer, the transported scalar $\tilde{R}e_{\theta t}$ converges towards the local fitted $Re_{\theta t}$ value, while inside the boundary layer, $\tilde{R}e_{\theta t}$ depends on the transport diffusion of the equation. The empirical correlation used to calculate $Re_{\theta t}$ can be defined as:

(38) $$Re_{\theta t}=\left[1173.51-589.428Tu+\frac{0.2196}{Tu^{2}}\right]F\left(\lambda_{\theta }\right),\;\;\;\;Tu\leq 1.3$$

(39) $$Re_{\theta t}=331.50{\left[Tu-0.5658\right]}^{-0.671}F\left(\lambda_{\theta }\right),\;\;\;\;Tu>1.3$$

(40) $$F\left(\lambda_{\theta }\right)=1-\left[-12.986\lambda_{\theta }-123.66\lambda_{\theta }^{2}-405.689\lambda_{\theta }^{3}\right]e^{-{\left[\frac{Tu}{1.5}\right]}^{1.5}},\;\;\;\;\lambda_{\theta }\leq 0$$

(41) $$F\left(\lambda_{\theta }\right)=1+0.275\left[1-e^{\left[-35.0\lambda_{\theta }\right]}\right]e^{\left[\frac{-Tu}{0.5}\right]},\;\;\;\;\lambda_{\theta }>0$$

(42) $$\lambda_{\theta }=\frac{\rho \theta^{2}}{\mu }\frac{dU}{ds}$$

(43) $$Tu=100\frac{\sqrt{2k/3}}{U}$$

where $\lambda_{\theta }$ is the pressure gradient parameter, which will be further explained and modified in the subsequent discussion. $\frac{dU}{ds}$ is the acceleration along the flow direction, which can be calculated by first taking the derivatives of U in the x, y, z directions and then superimposing the derivatives along the flow direction:

(44) $$U={\left({U_{x}}^{2}+{U_{y}}^{2}+{U_{z}}^{2}\right)}^{\frac{1}{2}}$$

(45) $$\frac{dU}{ds}=\left[(U_{x}/U)\frac{dU}{dx}+(U_{y}/U)\frac{dU}{dy}+(U_{z}/U)\frac{dU}{dz}\right]$$

The $\gamma \text{–}Re_{\theta t}$ transition model interacts with the k–ω SST turbulence model as follows:

(46) $$\frac{\partial }{\partial t}(\rho k)+\frac{\partial }{\partial x_{j}}\left(\rho U_{j}k\right)={\tilde{P}}_{k}-{\tilde{D}}_{k}+\frac{\partial }{\partial x_{j}}\left[\left(\mu +\sigma_{k}\mu_{t}\right)\frac{\partial k}{\partial x_{j}}\right]$$

(47) $$\frac{\partial }{\partial t}(\rho \omega )+\frac{\partial }{\partial x_{j}}\left(\rho U_{j}\omega \right)=\alpha \frac{P_{k}}{v_{t}}-D_{\omega }+Cd_{\omega }+\frac{\partial }{\partial x_{j}}\left[\left(\mu +\sigma_{k}\mu_{t}\right)\frac{\partial \omega }{\partial x_{j}}\right]$$

(48) $${\tilde{P}}_{k}=\gamma_{eff}P_{k}$$

(49) $${\tilde{D}}_{k}=\min\left[\max\left(\gamma_{eff},0.1\right),1.0\right]D_{k}$$

where $P_{k}$ and $D_{k}$ represent the production and destruction terms, respectively, in the turbulent kinetic energy equation within the original k–ω SST turbulence model [45]. $\gamma_{eff}$ is an effective intermittency after modification, which can be derived through the following equation:

(50) $$\gamma_{sep}=\min\left(s_{1}\max\left[0,\left(\frac{Re_{v}}{3.235Re_{\theta c}}\right)-1\right]F_{reattach},2\right)F_{\theta t}s_{1}=2$$

(51) $$F_{reattach}=e^{-{\left(\frac{R_{T}}{20}\right)}^{4}}$$

(52) $$\gamma_{eff}=\max\left(\gamma ,\gamma_{sep}\right)$$

The explicit details regarding the modeling functions and model parameters have been omitted for brevity’s sake, and the comprehensive elucidation of the k–ω SST turbulence model and $\gamma \text{–}Re_{\theta t}$ transition model can be found in Refs. [7,45], respectively.

### 2.3. High-Speed Modification Methods

This section aims to improve the applicability of the Langtry–Menter transition model in conjunction with the GKS method for hypersonic flow simulations. Here, we present three crucial high-speed modification techniques for the turbulence/transition model.

#### 2.3.1. Hypersonic Crossflow Extension

In the external boundary layer of a practical three-dimensional aircraft, the near-wall region exhibits a transverse component perpendicular to the main flow direction, commonly known as crossflow, due to the influence of transverse pressure gradients. The presence of an inflection point in the crossflow velocity profile leads to the occurrence of a cross-flow-induced transition, resulting from the instability it generates. Addressing this complex phenomenon, Langtry et al. [46] proposed an innovative localized low-speed crossflow prediction method that can be effectively integrated into the transition model. Zhou et al. [47] introduced a crossflow time scale into the transition model by calculating boundary layer parameters using grid preprocessing to enhance the model’s capability in predicting hypersonic transition induced by crossflow instability. Qiao et al. [48] developed a localized transition criterion for predicting the instability of three-dimensional profile crossflow based on the analysis of the compressible Falkner–Skan–Cooke similar solution. In this study, we refer to the low-speed crossflow transition method proposed by Langtry [46] and the high-speed crossflow transition criterion method proposed by Xiang [24], and introduce the following innovations based on these approaches: (1) Introducing a temporal scale term to modify the source term of the original high-speed crossflow transition criterion. (2) Transforming the original fixed roughness reference length into the variable transition momentum thickness. This crossflow correction method is integrated into our in-house CFD program to enable the prediction of three-dimensional hypersonic crossflow transition.

The approach for implementing localized crossflow correction in the Langtry–Menter transition model generally involves a four-step procedure that relies on assessing crossflow strength and surface roughness. Firstly, the formal definition of localized streamwise vorticity, denoted as $\Omega_{Streamwise}$, is established.

(53) $$\vec{U}=\left(\begin{array}{l} \frac{U_{x}}{\sqrt{{U_{x}}^{2}+{U_{y}}^{2}+{U_{z}}^{2}}},\\ \frac{U_{y}}{\sqrt{{U_{x}}^{2}+{U_{y}}^{2}+{U_{z}}^{2}}},\\ \frac{U_{z}}{\sqrt{{U_{x}}^{2}+{U_{y}}^{2}+{U_{z}}^{2}}} \end{array}\right)$$

(54) $$\vec{\Omega }=\left(\frac{\partial U_{z}}{\partial y}-\frac{\partial U_{y}}{\partial z},\frac{\partial U_{x}}{\partial z}-\frac{\partial U_{z}}{\partial x},\frac{\partial U_{y}}{\partial x}-\frac{\partial U_{x}}{\partial y}\right)$$

(55) $$\Omega_{Streamwise}=\left|\vec{U}\cdot \vec{\Omega }\right|$$

where $\vec{U}$ is the unit velocity vector and the concept of streamwise vorticity $\Omega_{Streamwise}$ is an indicator of the local crossflow strength in the boundary layer. The next step is to non-dimensionalize $\Omega_{Streamwise}$ into a measure of the crossflow strength, where the non-dimensional crossflow strength is defined as follows:

(56) $$H_{Crossflow}=\frac{y\Omega_{Streamwise}}{U}$$

where $H_{Crossflow}$ is a constant close to the wall, which reaches a maximum within the central region of the laminar boundary layer and then goes to zero at the boundary layer edge. The third step is to establish a high-speed crossflow criterion. The transition criterion uses a crossflow momentum thickness Reynolds number $Re_{HCF}$ as the fundamental parameter for assessing the occurrence of transition, while the non-dimensional treatment of surface roughness height is based on momentum thickness. By utilizing the theoretical frameworks of the low-speed and high-speed crossflow transition criteria, a novel empirical criterion for high-speed crossflow transition is established as follows, which elucidates the relationship between the crossflow momentum thickness Reynolds number $Re_{HCF}$ and the non-dimensional roughness $\frac{h}{\theta_{t}}$.

(57) $$Re_{HCF}=C_{1}\mathrm{Ln}\left(\frac{h}{\theta_{t}}\right)+C_{2}+f\left(H_{crossflow}\right)=\frac{\theta_{t}\rho \left(\frac{U}{0.82}\right)}{\mu }$$

(58) $$f\left(H_{crossflow}\right)=6000\left|0.1066-\Delta H_{crossflow}\right|+50000{\left(0.1066-\Delta H_{crossflow}\right)}^{2}$$

(59) $$\Delta H_{crossflow}=H_{crossflow}\left(1.0+\min\left[1.0\frac{\mu_{t}}{\mu },0.4\right]\right)$$

where $C_{1}=-14.7$ and $C_{2}=296.77$. $\theta_{t}$ represents momentum thickness, which can also be interpreted as the characteristic thickness of the boundary layer. The significant distinction between the correlation proposed in this study and the correlation presented in reference [24] lies in the substitution of a fixed reference length with the variable momentum thickness that effectively represents the local flow field characteristics. This modification imparts universality to the concept of surface relative roughness $\frac{h}{\theta_{t}}$, ensuring its applicability in various scenarios and enabling a more accurate depiction of its physical significance. It should be noted that the momentum thickness appears on both the left and right sides of Equation (57), necessitating the use of an iterative method such as the Newton method to solve for it. On the left-hand side of Equation (57), the velocity magnitude U is divided by 0.82 in order to compensate for the approximate difference between the freestream velocity and the local velocity within the boundary layer, where the vorticity Reynolds number reaches its maximum and transition starts. $\Delta H_{crossflow}$ is the crossflow strength shift term, which plays a crucial role in predicting the transition location accurately. When the transition occurs, the increase in eddy viscosity alters the velocity profile, causing a decrease in the value of $H_{crossflow}$. If this effect is not accounted for, it can lead to incorrect predictions of the transition location. However, this issue is mitigated by the inclusion of the viscosity ratio term $\frac{\mu_{t}}{\mu }$ in Equation (59), which cancels out this influence and ensures accurate predictions of the transition location.

In the laminar boundary layer, the crossflow strength is only a meaningful quantity. Hence, we introduced a novel crossflow sink term, denoted as $D_{HCF}$, into the $\tilde{R}e_{\theta t}$ transport equation, consistent with the form of Langtry’s crossflow extension [46]. The sink term is formulated as follows:

(60) $$\frac{\partial \left(\rho \tilde{R}e_{\theta t}\right)}{\partial t}+\frac{\partial \left(\rho U_{j}\tilde{R}e_{\theta t}\right)}{\partial x_{j}}=P_{\theta t}+D_{HCF}+\frac{\partial }{\partial x_{j}}\left[\sigma_{\theta t}\left(\mu +\mu_{t}\right)\frac{\partial \tilde{R}e_{\theta t}}{\partial x_{j}}\right]$$

(61) $$D_{HCF}=c_{HCF}\frac{\rho }{c_{t}t}\min\left(Re_{HCF}-\tilde{R}e_{\theta t},0\right)F_{\theta t}$$

(62) $$t=\min\left(\frac{500\mu }{\rho U^{2}},\frac{\rho L^{2}}{\left(\mu +\mu_{t}\right)}\right)$$

where the crossflow coefficient $c_{HCF}=0.018$. The $F_{\theta t}$ term effectively constrains the crossflow sink term $D_{HCF}$ to operate exclusively within the confines of the laminar boundary layer. Additionally, the activation of the $D_{HCF}$ term occurs only when the crossflow correlation $Re_{HCF}$ falls below the transported onset criteria $\tilde{R}e_{\theta t}$. Compared with the destruction term proposed in reference [23], this study’s improvement lies in the addition of a time-scale term in the sink term combined with an adjustable parameter $c_{t}$. This advancement allows for the sink term to be embedded into Equation (60) in a manner that ensures dimensional consistency with the transport equation. Furthermore, it achieves formal consistency with Langtry’s low-speed crossflow sink term [46].

#### 2.3.2. The Pressure–Dilatation Correlation

The formulation of the standard k–ω turbulence model equations for turbulent kinetic energy and the dissipation rate is originally derived under the assumption of incompressibility. This assumption implies that the eddy viscosity model inherently assumes zero velocity divergence. However, when considering the compressibility of the fluid, the assumption of zero velocity divergence is no longer valid, necessitating a transition from Reynolds averaging to Favre averaging. As a result, modifications are made to the formulation of the turbulence model, leading to the generation of new terms. The effects arising from non-zero velocity divergence in compressible turbulence are referred to as dilatation compressibility effects, and the modeling of the newly generated terms is known as the dilatation compressibility correction. Taking into account compressibility, the turbulent kinetic energy equation can be re-derived as follows:

(63) $$\begin{array}{l} \frac{\partial }{\partial t}(\bar{\rho }k)+\frac{\partial }{\partial x_{j}}\left(\bar{\rho }{\tilde{U}}_{j}k\right)=\bar{\rho }\tau_{ij}\frac{\partial {\tilde{U}}_{i}}{\partial x_{j}}-\bar{\rho }\epsilon +\\ \frac{\partial }{\partial x_{j}}\left[\bar{t_{ji}U_{i}^{\prime \prime }}-\bar{\rho U_{j}^{\prime \prime }\frac{1}{2}U_{i}^{\prime \prime }U_{i}^{\prime \prime }}-\bar{p^{\prime }U_{j}^{\prime \prime }}\right]-\bar{U_{i}^{\prime \prime }}\frac{\partial p}{\partial x_{i}}+\bar{p^{\prime }\frac{\partial U_{i}^{\prime \prime }}{\partial x_{i}}} \end{array}$$

The two newly generated terms are identified as the pressure work term $-\bar{U_{i}^{\prime \prime }}\frac{\partial p}{\partial x_{i}}$ and the pressure–dilatation term $\bar{p^{\prime }\frac{\partial U_{i}^{\prime \prime }}{\partial x_{i}}}$. The magnitude of $\bar{U_{i}^{\prime \prime }}$ is typically negligible and often disregarded. In this study, the influence of $-\bar{U_{i}^{\prime \prime }}\frac{\partial p}{\partial x_{i}}$ is not taken into account. Only the pressure–dilatation term $\bar{p^{\prime }\frac{\partial U_{i}^{\prime \prime }}{\partial x_{i}}}$ is modeled. $\bar{p^{\prime }\frac{\partial U_{i}^{\prime \prime }}{\partial x_{i}}}$ represents the correlation between fluctuating pressure and fluctuating velocity divergence, performing a significant aspect that manifests the compressibility effects in the flow.

The turbulent kinetic energy dissipation rate in high-speed compressible turbulence can be considered as the summation of two distinct components: the incompressible dissipation rate $\varepsilon_{s}$ and the compressible dissipation rate $\varepsilon_{d}$ [49].

(64) $$\bar{\rho }\varepsilon =\bar{\rho }\varepsilon_{s}+\bar{\rho }\varepsilon_{d}$$

It can be observed that the correction for compressibility mainly consists of two components: the compressible dissipation rate $\varepsilon_{d}$ and the pressure–dilatation term $\bar{p^{\prime }\frac{\partial U_{i}^{\prime \prime }}{\partial x_{i}}}$. The approaches proposed by Sarkar [50] have gained significant adoption in relation to these two aspects. Sarkar conducted a study on the decay of isotropic turbulence, investigating the relationship between compressibility and the turbulent Mach number. The findings revealed that the ratio of the compressible dissipation rate to the incompressible dissipation rate is directly proportional to the square of the turbulent Mach number. Consequently, a proposed model for the compressible dissipation rate is presented as follows:

(65) $$\varepsilon_{d}=\alpha_{1}\varepsilon_{s}M_{t}^{2}$$

where $M_{t}=\sqrt{2k}/a$ represents turbulent Mach number, a represents the local speed of sound, and $\alpha_{1}=1$ is the model coefficient. For the modeling of the pressure–dilatation term, pressure fluctuations are decomposed into rapidly varying and slowly evolving components. Drawing inspiration from the modeling concepts of the pressure–strain term in incompressible turbulence, a formulation for the pressure–dilatation term was established as follows:

(66) $$\bar{p^{\prime }\frac{\partial U_{i}^{\prime \prime }}{\partial x_{i}}}=-\alpha_{2}P_{k}M_{t}^{2}+\alpha_{3}\bar{\rho }\varepsilon_{s}M_{t}^{2}$$

in which $P_{k}$ is the production of the turbulent energy equation. $\alpha_{2}$ and $\alpha_{3}$ are model coefficients, taking values of −0.15 and 0.2, respectively.

#### 2.3.3. Pressure Gradient Parameter Correction

The boundary layer transition is highly sensitive to the streamwise pressure gradient. In the $\gamma \text{–}Re_{\theta t}$ original transition model, the empirical formula for calculating the $Re_{\theta t}$ contains the parameter $\lambda_{\theta }$ related to the pressure gradient information, defined as Equation (42), where $\frac{dU}{ds}$ is the flow velocity gradient, θ is the momentum thickness, and μ is the molecular viscosity. The pressure gradient parameter $\lambda_{\theta }$ reflects the relationship between the pressure gradient, velocity gradient, and boundary layer thickness. The pressure gradient information is passed to $Re_{\theta t}$ via the parameter $\lambda_{\theta }$, subsequently influencing the momentum thickness Reynolds number transport equation. Due to an increase in the Mach number, the boundary layer thickness escalates, altering the relationship between the pressure gradient and the velocity gradient from that in low-speed flow to that in hypersonic flow. The change in the streamwise pressure gradient brought about by varying boundary layer thickness has a significant effect on the hypersonic transition. The impact of a high Mach number should thus be taken into consideration in simulation calculations for hypersonic transition. In this study, referring to the modification method proposed by Zhang et al. [21], we derive an expression for the pressure gradient parameter after the correction for high Mach numbers.

(67) $$\lambda_{\theta_{-}new}=\lambda_{\theta }\left(1+\frac{\gamma \prime -1}{2}M_{e}^{2}\right)b_{2}$$

where γ′ represents the specific heat ratio, $M_{e}$ denotes the Mach number at the boundary layer edge, and $b_{2}$ is an adjustable coefficient.

## 3. Coupling Mechanism in the Mesoscopic Transition Prediction Method

**Figure 1:**
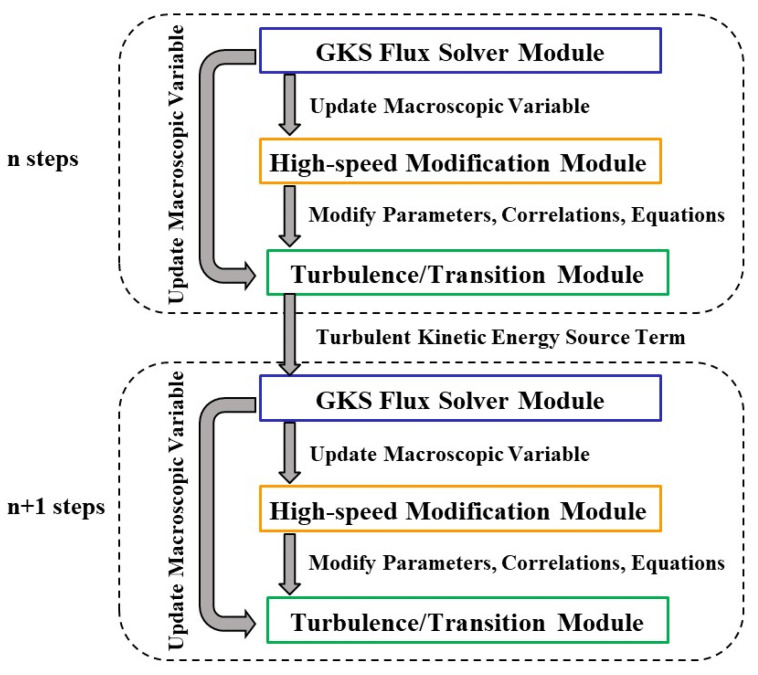
Program framework schematic diagram for mesoscopic methods in transition prediction.

The innovative contribution of this study lies in the integration of the Langtry–Menter transition model and its high-speed modifications with the GKS method, resulting in the development of a mesoscopic framework for transition prediction. Figure 1 provides a schematic representation of this integrated program. The proposed numerical scheme comprises three essential modules: (1) the GKS flux solver module, (2) the turbulence/transition module, and (3) the high-speed modification module. By incorporating the turbulent kinetic energy term into the Maxwellian velocity distribution function and utilizing the turbulence transition model, the source term for turbulent kinetic energy is computed, establishing a coupling between the GKS method and the transition equations. The macroscopic variables obtained from the GKS solver are fed back into the modification module, facilitating the integration of the modification module into the mesoscopic GKS method. Within the modification module, three high-speed modification techniques, either directly or indirectly through the transition onset momentum thickness Reynolds number and intermittency factor, influence the source term for turbulent kinetic energy, consequently affecting the GKS distribution function. In other words, the three aforementioned high-speed modification methods have a direct or indirect impact on the velocity distribution function. This entire process realizes the coupling of the Langtry–Menter transition model and its high-speed modifications with the GKS method, integrating the crossflow effect, compressibility effect, and pressure gradient modification at the mesoscopic level. The groundbreaking aspect of this approach lies in the coupling of molecular motions and eddy movements, encompassing a broader range of physical mechanisms compared with traditional methods.

## 4. Results and Discussion

This section aims to comprehensively evaluate the performance and accuracy of the existing mesoscopic numerical methods used for the prediction of hypersonic transition. To achieve this objective, simulations are conducted on well-established hypersonic transitional flow cases, including two-dimensional flat plates and three-dimensional cones with zero and small angles of attack (AOA). Additionally, previous research [51] has identified three additional models, namely, HiFIRE-1, HiFIRE-5, and a flared-cone model, which warrant further exploration and validation. Each of these models possesses unique characteristics and effectively encapsulates the physical essence of transition-related issues encountered in engineering applications. By using our in-house CFD code, we extend our investigation to include the HiFIRE-1 and HiFIRE-5 models, thereby validating the efficacy of mesoscopic numerical methods in predicting hypersonic transition within practical engineering contexts. This expanded analysis serves as a valuable bridge between academic research endeavors and the technical requirements of engineering applications.

### 4.1. Supersonic Adiabatic Flat Plate with Ma_∞_ = 4.5

**Figure 2:**
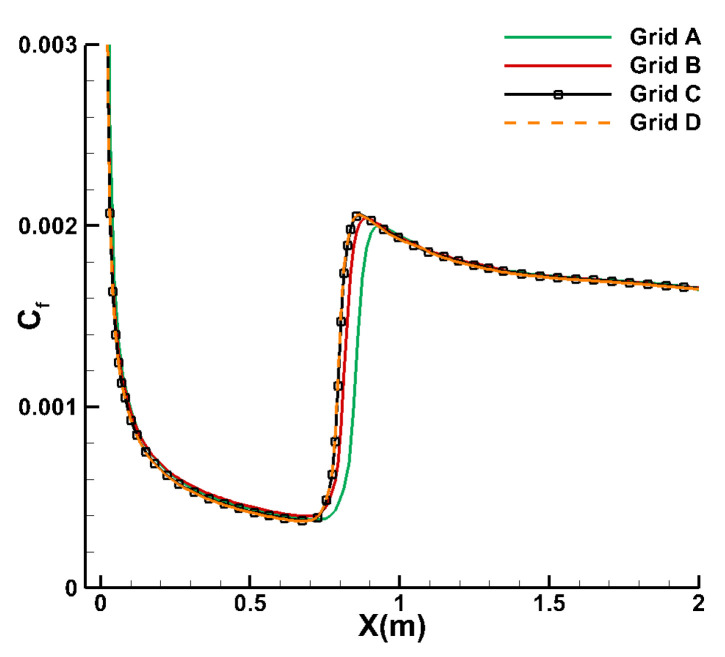
Comparison of skin friction coefficient distribution curves for different grids.

**Figure 3:**
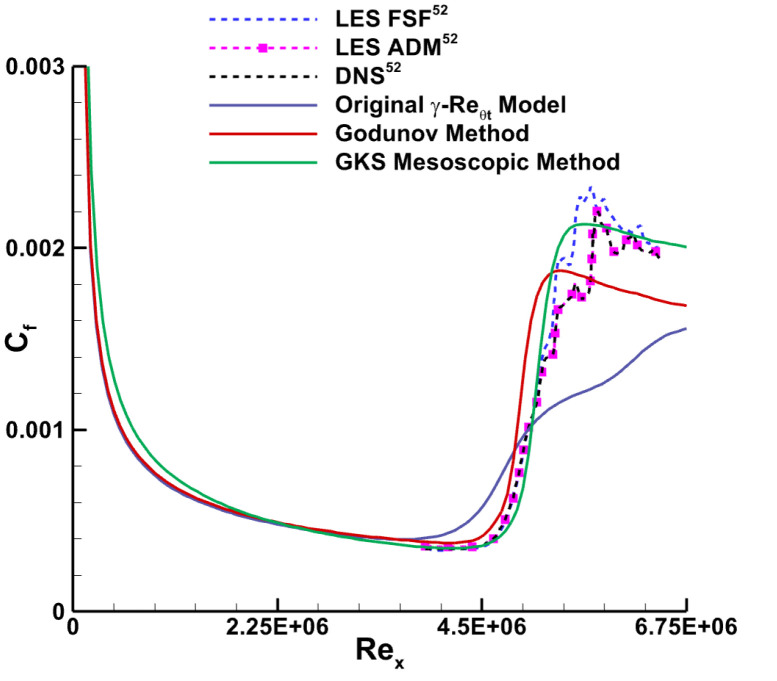
Distribution of the coefficient of skin friction for an adiabatic hypersonic flat plate.

The validation case discussed in this subsection focuses on the adiabatic wall of a supersonic flat plate. The inflow condition is characterized by a Mach number of 4.5, a unit Reynolds number of $Re_{\infty }=6.433\times 10^{6}/m$, and an inflow temperature of $T_{\infty }=61.1\;K$. A freestream turbulence intensity of 0.1% is set, and the viscosity ratio is fixed at 0.1. Four sets of grids with varying densities were generated, and their properties are summarized in Table 1. A grid independence verification was conducted, and the comparison of wall friction coefficient distributions obtained from these four grids is presented in Figure 2. The frictional resistance curves calculated using different grids exhibit generally consistent trends, with minor deviations observed in the results from Grid A. These discrepancies are primarily manifested in the transition onset and offset locations. Notably, the numerical results obtained from Grid C and Grid D overlap completely, indicating grid convergence and achieving grid independence in our computations. The y^+^ value for Grid C is 0.25, which is less than 1. Figure 3 showcases the numerical results of the skin friction coefficient distribution obtained using various computational methods. The blue solid curve corresponds to results from the original transition model, which proves ineffective in supersonic scenarios. The red solid line represents simulations incorporating the high-speed modification module while still utilizing the Godunov scheme, which is essentially a traditional Riemann solver. For these simulations, the flux splitting technique uses the AUSMPW+ (Advection Up-stream Splitting Method by Pressure-based Weight functions) scheme, and reconstruction is performed using the MUSCL (Monotone Upstream-centered Schemes for Conservation Laws) method. The subsequent cases in this paper utilizing the Godunov method also adopt these schemes. The green solid line depicts results from the newly proposed GKS mesoscopic method in this study. The black dashed line, blue dashed line, and pink dashed line represent the DNS results, LES Approximate-Deconvolution Model (ADM) results, and LES Filtered-Structure Function (FSF) results, respectively. These three high-order and high-precision numerical results, sourced from the existing literature [52], serve as benchmark standards for evaluating the novel methodology presented in this study. In comparison with the Godunov technique, the newly proposed mesoscopic method exhibits a notably improved level of agreement with the DNS data [52] and the results [52] obtained from two LES formulations.

### 4.2. Hypersonic Transition on Cones

**Figure 4:**
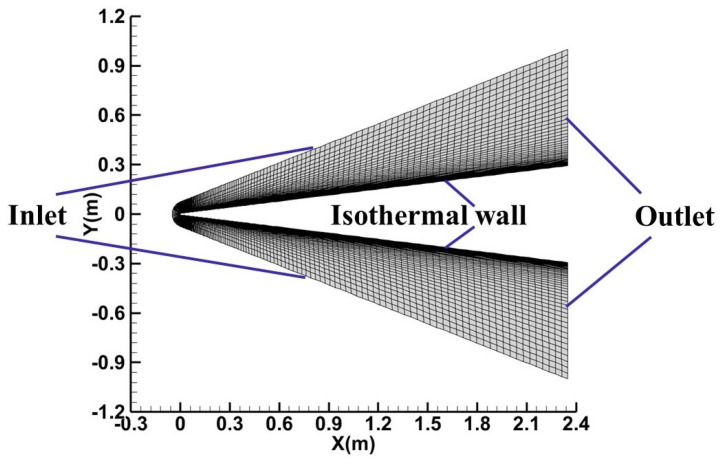
Schematic of the computational mesh and boundary conditions.

**Figure 5:**
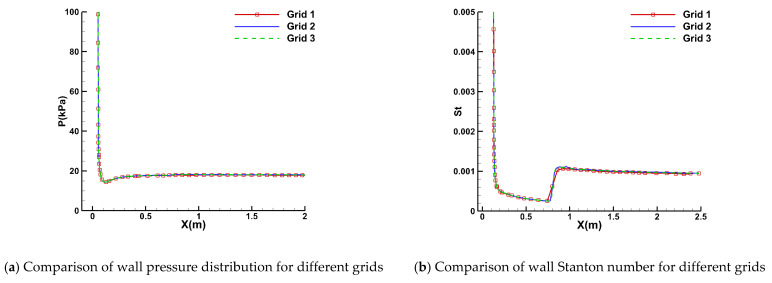
Grid independence study.

This subsection conducts a comprehensive numerical analysis of boundary-layer transition flow over hypersonic cones, consisting of two distinct series of cases. Each case is characterized by specific experimental conditions, nose radiuses, and AOAs. These variations allow for a thorough evaluation of the novel GKS mesoscopic transition prediction method proposed in this study, as well as the effectiveness of the modifications for hypersonic applications. The first series of hypersonic cone transition experiments were conducted using the LENS-I hypervelocity shock tunnel [53]. The experimental model used a 2.35-m-long cone with a half-angle of 7 degrees, accompanied by two variants featuring nose radii of 5.0 mm and 6.35 mm. Table 2 presents the detailed experimental conditions for this first series of hypersonic cone cases, all of which were tested at zero AOA. The primary hypersonic flow regimes explored encompass velocities of Mach 7 and Mach 10. A grid independence study was conducted using a half-model grid, involving three sets of grids with varying densities, under the computational condition of Case Ⅰ. The characteristics of these three grids are detailed in Table 3. Figure 4 illustrates the symmetry plane of the computational mesh and the corresponding boundary conditions. The aerodynamic and thermal computational results for these grids are depicted in Figure 5. The wall pressure distribution for all three grid sets was found to be identical. The wall Stanton numbers calculated for Grids 2 and 3 almost perfectly overlapped, while Grid 1 exhibited a slight deviation in the thermal simulation results near the transition location. Based on these findings, Grid 2 was determined to satisfy the grid convergence criteria and was thus selected for subsequent computational analyses. The calculated y^+^ value for Grid 2 approximates 0.36, which is less than 1.

**Figure 6:**
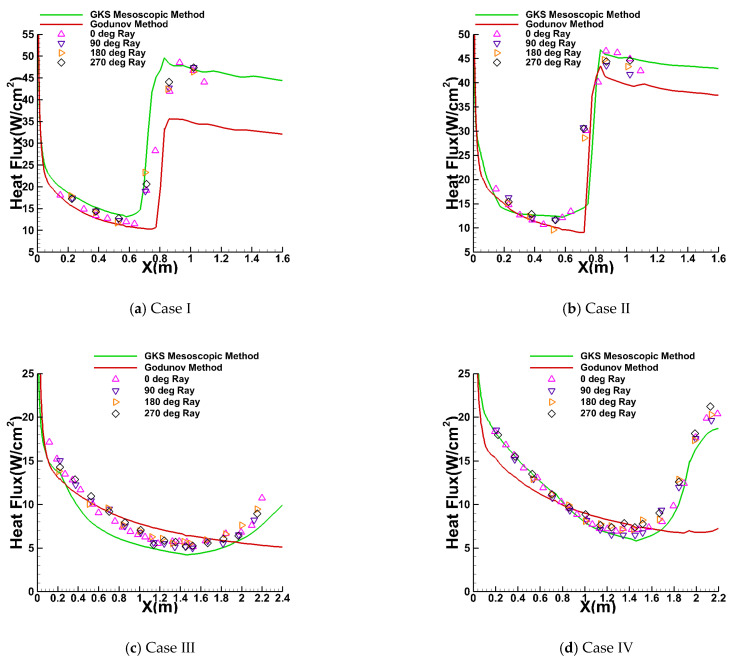
Comparison of the heat flux predictions and the experimental results for the first series of cones.

Figure 6 depicts the heat flux predictions for four cone cases obtained through the utilization of both the GKS-coupled mesoscopic method and the classical Godunov method, alongside the corresponding experimental results. The experimental data [54], represented by multicolored symbols, was acquired by using thin-film heat transfer gauges strategically positioned at 0°, 90°, 180°, and 270° around the cone model. The numerical results obtained using the GKS mesoscopic method and the Godunov method are depicted by green and red solid lines, respectively. An analysis of the results reveals that, for Case Ⅰ and Case Ⅱ, both the newly developed coupled mesoscopic method and the traditional Godunov method accurately predict the location of transition onset. However, in the turbulent flow region, the coupled mesoscopic method demonstrates superior prediction accuracy compared with the traditional Godunov method. Conversely, in Case Ⅲ and Case Ⅳ, characterized by a higher free-stream Mach number of 10, only the mesoscopic method successfully predicts the onset location of the transition. Remarkably, the GKS numerical results agree well with the experimental data, effectively capturing the heat flux distribution and accurately predicting the curve’s trend. In contrast, the Godunov method proves ineffective in these higher-speed cases, as its predicted trends run counter to the real situation. Based on these findings, it is evident that the current method excels in predicting three-dimensional hypersonic transition flows. This is attributed to its consideration of microscopic molecular motion, where turbulent kinetic energy and high-speed modification methods are added to the velocity distribution function.

**Figure 7:**
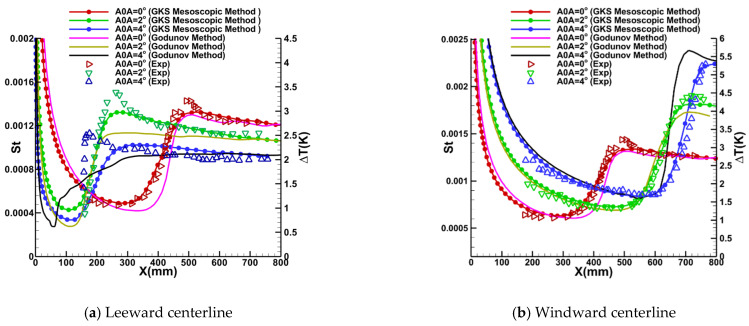
Comparison of the transition onset for supersonic flow over the sharp cone at different AOAs.

The second series of supersonic transition cases focuses on a sharp cone at varying angles of attack. These experiments were conducted at the Φ = 1 m hypersonic wind tunnel [55]. The test model used in these experiments is a seven-half-angle cone with a nose radius of 0.05 mm and an axial length of 800 mm. The experiments were performed under three different angles of attack: 0°, 2°, and 4°. The angle of attack induces pressure disparities between the windward and leeward surfaces of the cone. A lateral flow velocity perpendicular to the principal flow direction is generated as the flow moves from the windward side to the leeward side. This lateral flow velocity induces crossflow instability, thereby leading to transition. The freestream conditions for the simulations consist of a freestream Mach number $Ma_{\infty }=6$, a freestream unit Reynolds number $Re_{\infty }=1\times 10^{7}/m$, an inflow temperature $T_{\infty }=57.81\;K$, and an isothermal wall condition $T_{wall}=300\;K$. The turbulence intensity is maintained at 0.6%, with a viscosity ratio of 10. A computational grid with the near-wall resolution y^+^ ≈ 0.35 and dimensions of 213 × 130 × 32 cells in the streamwise, normal, and circumferential directions, respectively, is utilized for the numerical simulations, which is confirmed through a grid independence study. Figure 7 illustrates the comparative analysis of the surface Stanton number as predicted by our current GKS-coupled mesoscopic method (represented by dot-line curves), the conventional Godunov method (represented by solid curves), and the experimental results [55] (indicated by symbols) along the leeward and windward centerline. The different colors in Figure 7 correspond to different AOAs. The experimental results are the instantaneous temperature rise data measured on the cone surface. The onset and termination of boundary layer transition are identified by the lowest and highest points of the temperature rise, respectively. The Stanton number is approximated to be proportional to the temperature rise, and the location of the transition onset is inferred from the distribution of the temperature rise. Thus, a direct comparison is made between the calculated Stanton number and the temperature rise data from the experimental set-up while ignoring their unit dissimilarities.

It is elucidated in Figure 7 that at 0^o^ AOA, the numerical results derived from both the GKS mesoscopic method and the Godunov method exhibit congruence with the experimental data, and the location of transition onset location can be identified as the Stanton number departs from its laminar value. However, the GKS mesoscopic method demonstrates superior predictive capability for the transition onset position compared to the Godunov method. In two test cases involving non-zero AOAs, the GKS mesoscopic method outperforms the Godunov method in terms of predicting the transition onset position and the turbulent portion. It is worth noting that the crossflow modification module is implemented in both computational schemes for these non-zero AOAs. This superior performance can be attributed to the integration of velocity macroscopic variables, updated from the GKS framework, into the high-speed crossflow correction method. The influence of crossflow modification benefits on turbulent energy subsequently impacts the distribution function, leading to the integration of the crossflow modification’s advantages into the mesoscopic method. Consequently, both the physical mechanisms and correction effects considered in this approach significantly surpass those of the conventional Godunov method. Additionally, Figure 7 reveals that along the windward centerline, the Stanton number gradually increases as the AOA increases, accompanied by a downstream shift in the transition onset location. Conversely, on the leeward side, an increase in AOA leads to a decrease in the Stanton number, resulting in an upstream shift in the transition onset location. These findings provide compelling evidence demonstrating the capability of the GKS mesoscopic method to accurately simulate the impact of AOA on the transition onset locations along the windward and leeward sides of the cone.

### 4.3. HIFiRE-1 Hypersonic Flight

**Figure 8:**
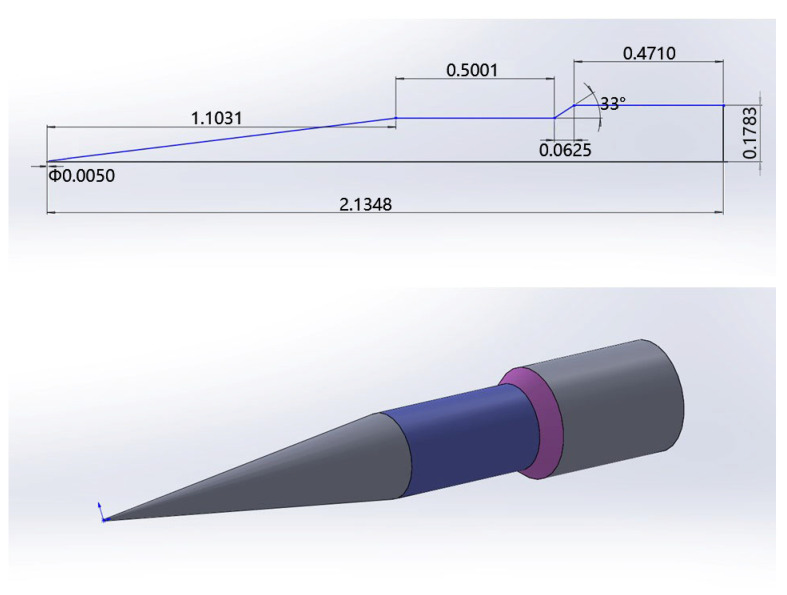
HIFiRE-1 payload configuration (dimensions in m).

The HIFiRE program [56] is a collaborative research endeavor carried out by the United States Air Force Research Laboratory (AFRL) and the Australian Defence Science and Technology Organisation (DSTO). The primary objective of the HIFiRE-1 [57] flight experiment was to obtain measurements of hypersonic boundary-layer transition and shock boundary-layer interactions that are challenging to assess solely using ground-based testing. The payload configuration for the HIFiRE-1 flight vehicle, as depicted in Figure 8, consists of three unique geometric segments: a cone, a cylinder, and a flare. The cone is 1.1 m long, featuring a seven-degree half angle and a nose bluntness characterized by a radius of 2.5 mm. Adjacent to the conical section, a cylindrical element with a length of 0.5 m serves as a transitional region between the cone and the flare. The rear section of the payload incorporates a flare with an angle of 33 degrees and a diameter of 0.356 m. In this subsection, we conduct numerical simulations to analyze the hypersonic transition flow of HIFiRE-1 at four specific time intervals throughout its flight trajectory. The flight conditions for each instance are presented in Table 4. Given that the experiment involves a real flight in the atmosphere, the turbulence intensity is set at $Tu_{\infty }=0.1\%$, and the wall temperature is maintained at $T_{wall}=350\;K$. We adopt a half-model computational grid with dimensions of 296 × 120 × 48 cells in the streamwise, normal, and circumferential directions, respectively, and set the spacing of the first grid to 5 × 10^−7^ m, which means y^+^ ≈ 0.26. The grid resolution above is carefully chosen to maintain accuracy and reliability in the simulations, ensuring proper capturing of the boundary-layer transition phenomena.

**Figure 9:**
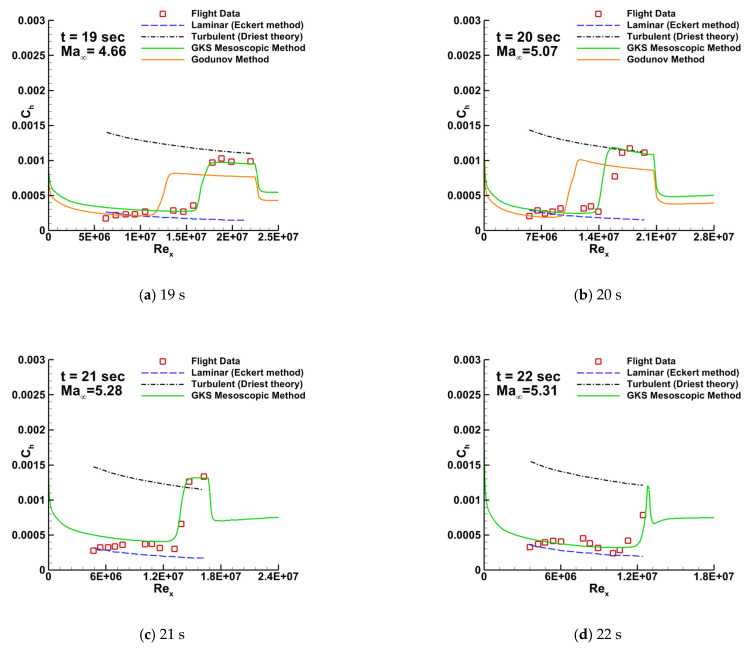
Comparison of the heat transfer coefficient for HIFiRE-1 at four distinct flight moments obtained by different methods.

Figure 9 presents the distribution of the heat transfer coefficient along the surface between t = 19 and 22 s. The full turbulence prediction is based on the van Driest [58] theory, while the full laminar flow prediction uses the Eckert [59] method. For the transition prediction, both the GKS mesoscopic method and the conventional Godunov method are used. The results depicted in Figure 9 clearly demonstrate that the GKS mesoscopic method exhibits superior agreement with the experimental flight data [57] in terms of transitional position and wall heat flux simulation. Although the standard Godunov method proves effective in simulating the laminar section, it falls short of accurately predicting the onset location of the transition and the turbulent zone. This limitation becomes more pronounced when compared with the capabilities demonstrated by the GKS mesoscopic method.

**Figure 10:**
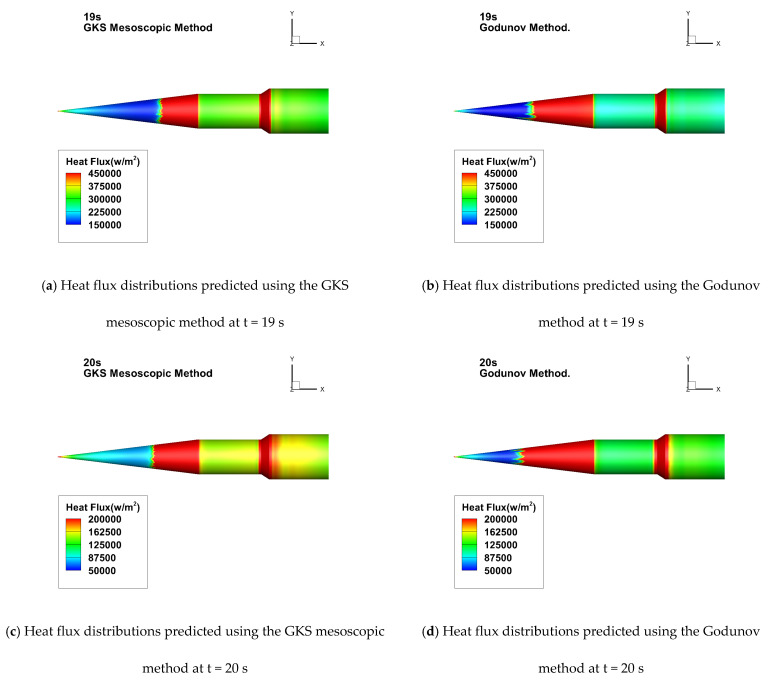
Comparison of wall heat flux distributions at two moments using two distinct methods.

Figure 10 presents the contour maps of the three-dimensional surface heat flux distribution for HIFiRE-1 at the 19-s and 20-s moments. The illustration reveals that the transition location delineated by the GKS mesoscopic method is characterized by a uniform and orderly depiction. In contrast, the conventional Godunov method yields numerical results in the transition zone that appear somewhat erratic and jagged. It is worth emphasizing that these simulation outcomes have undergone grid-independence verification, thereby eliminating the influence of grid-related factors. In light of these observations, it becomes apparent that the GKS mesoscopic method offers a more accurate and realistic prediction for hypersonic boundary layer transition. The predicted three-dimensional contour maps demonstrate a closer alignment with genuine physical depictions, displaying clear superiority.

In this section, we compare the computational efficiency of the Godunov method and the GKS mesoscopic method using the three-dimensional HIFiRE-1 case as a benchmark. The analysis was conducted under identical computational devices and environmental conditions. For the GKS mesoscopic method, each computational step in the HIFiRE-1 case took 1.73 s, and the simulation converged after 60,000 steps. In contrast, the Godunov method completed each step in 0.34 s and converged after 50,000 steps. These results indicate that the Godunov method is approximately five times faster than the GKS mesoscopic method in terms of computational speed. Furthermore, the GKS mesoscopic method exhibits slower convergence compared with the Godunov method. The observed disparity in computational performance can be attributed to the inherent complexity of the GKS flux computation, which, especially when coupled with a four-equation transition model, potentially impacts the overall computational efficiency.

### 4.4. HIFiRE-5

**Figure 11:**
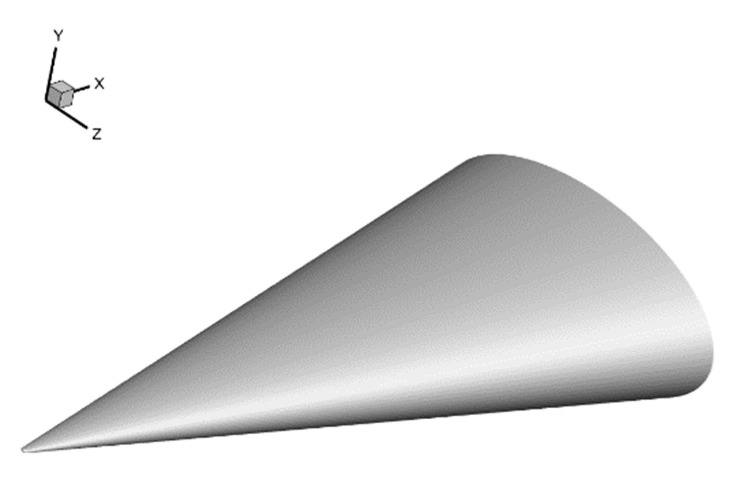
HIFiRE-5 model geometry.

**Figure 12:**
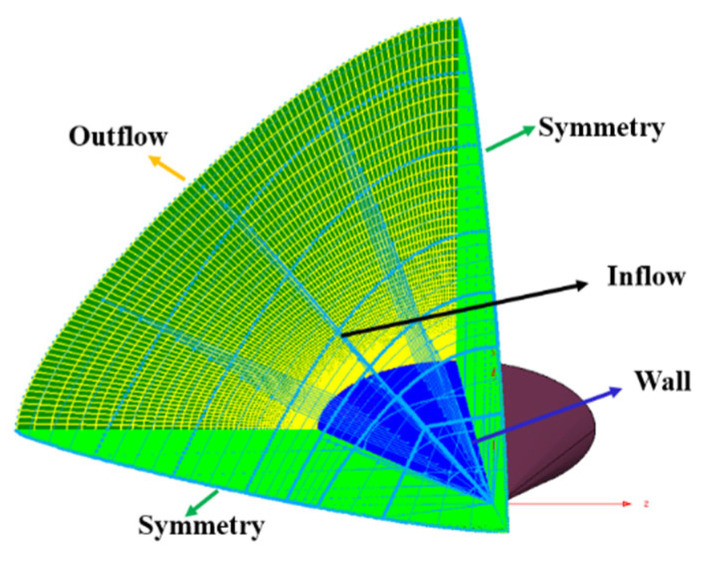
Schematic of the computational grid and boundary conditions.

The second HIFiRE test, known as HIFiRE-5 [60], is dedicated to studying boundary-layer transition on a three-dimensional (3D) body. In contrast to the axisymmetric configurations of HIFiRE-1, the 3D configurations introduce unique phenomena, such as leading-edge transition and crossflow instabilities. The HIFiRE-5 vehicle exhibits an elliptical cone shape with an aspect ratio of 2:1. It features a seven-degree half-angle on the minor axis, a nose tip radius of 2.5 mm, and a length of 0.86 m. In this study, a scaled-down model at 38.1% of the original size (shown in Figure 11) is utilized, and the experimental measurements were conducted in the Boeing/AFOSR Mach-6 Quiet Tunnel [61] under quiet flow. Notably, HIFiRE-5’s geometry ensures that the hypersonic crossflow transition occurs at a 0° angle of attack with a relatively strong spanwise pressure gradient. The specific test conditions used are presented in Table 5. Following a grid convergence analysis, a computational grid based on a one-fourth-scale model is used for the numerical simulations (shown in Figure 12). The grid dimensions are adjusted to 255 × 165 × 91 nodes in the streamwise, normal, and circumferential directions, respectively, with the first grid spacing set at 1 × 10^−6^ m, i.e., y^+^ ≈ 0.37 < 1.

**Figure 13:**
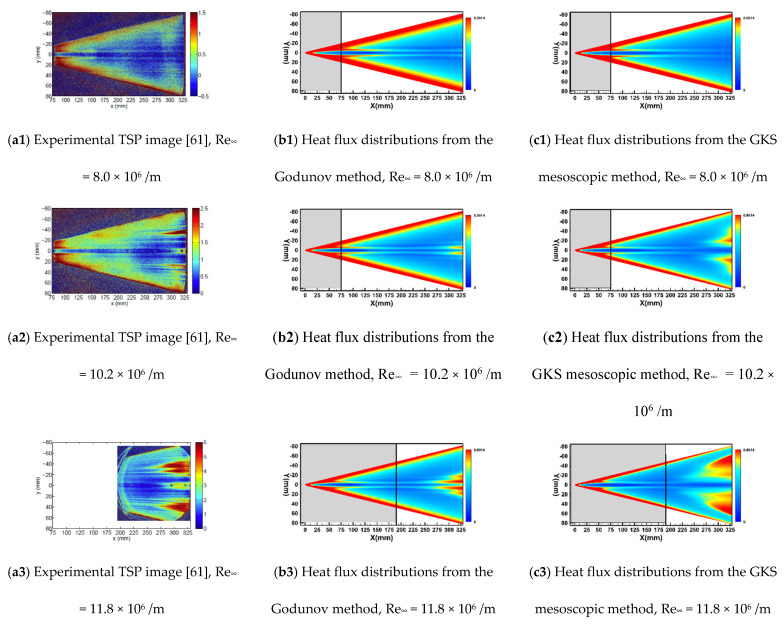
Transition distributions on the HIFiRE-5 surface.

Figure 13 illustrates the computational results of transition onset distributions at various Reynolds numbers on HIFiRE-5, along with corresponding experimental results obtained under quiet flow conditions. Figure 13a1–a3 displays the temperature-sensitive paint (TSP) contours obtained from quiet flow experiments conducted in Purdue’s Mach-6 Wind Tunnel [61] at corresponding Reynolds numbers. The computational outputs of wall heat flux, as predicted by the conventional Godunov method and the GKS mesoscopic method, are depicted in Figure 13b1–b3 and Figure 13c1–c3, respectively. Significant differences are evident between the predictive outcomes of the two computational schemes, despite their common implementation of high-speed crossflow modifications. Specifically, the conventional Godunov method tends to localize the transition region predominantly near the meridian, exhibiting a noticeable mismatch with observed experimental data. This misalignment highlights potential limitations in the predictive accuracy of the Godunov method. On the other hand, the GKS mesoscopic method demonstrates a marked improvement in alignment with experimental results, both in the streamwise and spanwise directions, regarding the simulated transition region. One notable advantage of the GKS mesoscopic approach is its ability to capture the phenomenon whereby the transition onset moves forward as the Reynolds number increases. Within the GKS mesoscopic framework, the macroscopic velocity quantities pertaining to the crossflow modification module undergo updates derived from the GKS solver. The integration of crossflow modification effects into the turbulent kinetic energy source term subsequently influences the velocity distribution function within the GKS solver. This integration elevates the correction mechanism for high-speed crossflows to a mesoscopic level, leading to a substantial enhancement in its corrective efficacy. It is precisely this coupling mechanism that endows the proposed method with the ability to improve the prediction of transition fronts, providing a more precise and authentic forecast for hypersonic boundary layer transition. The predicted three-dimensional contour maps closely align with genuine physical representations, showcasing a distinct advantage over conventional approaches.

## 5. Conclusions

In this paper, we present a novel GKS-coupled mesoscopic method aimed at predicting high-speed transition flow. The uniqueness of our approach lies in the integration of the Langtry–Menter transition model and its high-speed modifications within the GKS framework, culminating in an improved mesoscopic transition prediction method. Incorporating the turbulent kinetic energy term into the Maxwellian velocity distribution function and utilizing the turbulence transition model, we compute the source term for turbulent kinetic energy, effectively establishing a coupling between the GKS method and the transition model equations. The macroscopic variables derived from the GKS solver are seamlessly integrated into the three high-speed modification methods, facilitating a feedback loop that enhances the integration of the modifications within the mesoscopic GKS framework. Three high-speed modification techniques, either directly or indirectly through the transition onset momentum thickness Reynolds number and intermittency factor, precisely influence the source term for turbulent kinetic energy. This, in turn, has a profound impact on the GKS distribution function, effectively modulating the velocity distribution at the mesoscopic level. In essence, these high-speed modification methods exert a direct or indirect influence on the velocity distribution function, realizing a comprehensive integration of the Langtry–Menter transition model and its high-speed adaptations with the GKS methodology.

The efficacy and reliability of our newly proposed method were thoroughly examined and validated in a range of challenging test cases, including a supersonic adiabatic flat plate, hypersonic cones with small angles of attack, HIFiRE-1 hypersonic flight, and HIFiRE-5 wind tunnel tests. By benchmarking our numerical results against experimental data, we conducted a comprehensive comparative analysis between the GKS mesoscopic method and the conventional Godunov method, elucidating both the numerical outcomes and methodological principles. The GKS computational framework elevates the transition model and high-speed modifications to the mesoscopic level with multi-scale effects in space and time, encompassing a broader range of physical mechanisms than the conventional Godunov method. This innovative technique couples turbulent eddy motion with molecular motion, enhancing the predictive accuracy of transition flows. Furthermore, in challenging validation cases such as those involving cones with varying angles of attack and the HIFiRE series tests, the GKS mesoscopic method proves its robust practical simulation capabilities in three-dimensional high-speed transitional flows. This showcases its substantial potential for engineering applications and scientific research. The proposed method’s ability to accurately predict and capture transitional flow behavior under such challenging conditions underscores its validity and reliability, rendering it a valuable tool for future investigations.

## Figures and Tables

**Table 1:** Properties of the four sets of grids.

Mesh	Computational Mesh Dimensions (Streamwise × Normal)	First Layer Grid Spacing (m)
Grid A	175 × 120	1 × 10^−5^
Grid B	219 × 190	5 × 10^−6^
Grid C	259 × 200	1 × 10^−6^
Grid D	329 × 220	5 × 10^−7^

**Table 2:** Experimental conditions for the first series of hypersonic cone cases.

	*H*_0_ (MJ/kg)	*Ma_∞_*	*T_∞_* (K)	*ρ_∞_* (kg/m^3^)	*Re_∞_* (1/m)	*T_wall_* (K)	*R_n_* (mm)
Case I	2.62	7.15	231.1	0.07060	9.92 × 10^6^	301.0	5.0
Case II	2.09	6.58	214.0	0.12568	1.67 × 10^7^	301.3	5.0
Case III	4.49	9.91	206.71	0.0121	2.52 × 10^6^	295.61	6.35
Case IV	4.56	9.93	209.33	0.0160	3.33 × 10^6^	299.00	6.35

**Table 3:** Properties of three sets of straight cone grids.

Mesh	Computational Mesh Dimensions (Streamwise × Normal × Circumferential)	First Layer Grid Spacing (m)
Grid 1	104 × 60 × 13	1 × 10^−5^
Grid 2	186 × 101 × 31	1 × 10^−6^
Grid 3	226 × 150 × 37	5 × 10^−7^

**Table 4:** Flight conditions for the hifire-1 at four time intervals.

Time(s)	Altitude (km)	*P_∞_* (Pas)	*T_∞_* (K)	*Ma_∞_*	Unit *Re* (10^6^/m)
19.00	15.42	12317.90	205.30	4.66	20.58
20.00	16.75	9851.90	201.00	5.07	18.46
21.00	18.15	7753.70	199.20	5.28	15.28
22.00	19.58	6102.50	203.70	5.31	11.74

**Table 5:** Flow conditions for HIFiRE-5 under quiet flow.

Noise level	*Ma_∞_*	*Re_∞_* (10^6^/m)	*T_∞_* (K)	*T_wall_* (K)	*Tu_∞_*
Quiet flow	6.0	8.0, 10.2, 11.8	52.8	300	0.05%

## Data Availability

The data that support the findings of this study are available from the corresponding author upon reasonable request.
